# Case Report: Exploring the clinical spectrum of LGMD R27: insights from a case study with homozygous pathogenic variant in the *JAG2* gene

**DOI:** 10.3389/fped.2024.1414465

**Published:** 2024-11-22

**Authors:** Sergey Nikitin, Evgeniya Melnik, Inna Sharkova, Aysylu Murtazina, Olga Shchagina, Victoriia Zabnenkova, Vadim Tsargush, Elena Dadali, Sergey Kutsev

**Affiliations:** ^1^ Research Center for Medical Genetics, Moscow, Russia; ^2^ Center of Aviation Medicine, Moscow, Russia

**Keywords:** limb-girdle muscular dystrophy, LGMD R27, *JAG2*, case, severe clinical phenotype

## Abstract

Limb-girdle muscular dystrophies (LGMD) constitute a heterogeneous group of genetic disorders characterized by progressive muscle weakness and atrophy, predominantly affecting the muscles of the pelvic and shoulder girdles. LGMD R27, linked to biallelic pathogenic variants in the *JAG2* gene, was recently described, and to date, only 27 cases has been published in three reports. Here, we present two siblings exhibiting a severe clinical phenotype of LGMD R27, associated with a novel *JAG2* homozygous frameshift variant [c.3467_3470dup, p.(Pro1158AlafsTer22)] results in truncated protein with 21 amino acid substitution within the cytoplasmic domain of the Jagged2 protein.

## 1 Introduction

Limb-girdle muscular dystrophies (LGMDs) encompass a group of heterogeneous genetic disorders characterized by progressive muscle weakness and atrophy primarily affecting the pelvic and shoulder girdle muscles. Among the diverse array of LGMD subtypes, LGMD R27, linked to biallelic pathogenic variants in the *JAG2* gene, stands out as a rare and intriguing entity. To date, only 27 cases were published, 23 of them in a single study (1–3).

The *JAG2* gene encodes a transmembrane protein known as Jagged2, a ligand for the Notch1 receptor (4). Extensive research has established that Jagged2 protein is a component of the Notch signaling pathway. This pathway serves as a pivotal regulator of cell differentiation, facilitates intercellular communication, contributes to tissue homeostasis maintenance, and assumes a critical role in the myogenesis processes (5–9). Pathogenic variants within the *JAG2* gene reported in patients with LGMD R27 encompass a diverse spectrum, including missense, nonsense and frameshift mutations, each contributing to the heterogeneous clinical phenotype observed in affected individuals (1–3).

The clinical presentation of LGMD R27 is characterized by a gradual onset of muscle weakness and wasting, typically beginning in childhood or early adulthood and adolescence (1). Notably, all patients have a prevalent manifestation of diffuse muscular hypotonia and severe weakness, primarily affecting the pelvic girdle muscles. In addition, axial weakness is a significant hallmark, particularly impacting the muscles responsible for neck flexion. Furthermore, over half of LGMDR27 patients exhibit contractures in the knee, elbow, and ankle joints. Moreover, a subset of patients presents with various associated symptoms, including upper eyelid ptosis, scoliosis, cardiomyopathy, as well as an intellectual disability and autism spectrum disorders (1, 2). Progressive involvement of both proximal and distal muscles leads to significant functional impairment, impacting activities of daily living and diminishing quality of life (1, 3).

In this study, we present a case involving two siblings from a consanguineous family who harbor a novel homozygous pathogenic variant in the *JAG2* gene. The parents of the proband are first cousins of Tajik nationality and carry the detected variant in a heterozygous state.

## 2 Case description

The proband, a 14-year-old boy, is one of two affected siblings presenting with complaints of progressive muscle weakness, loss of ability to walk, limited range of motion in the arms, and joint contractures affecting both upper and lower limbs. He was born with a birth weight of 2.2 kg (−2.18 SD) from the first pregnancy. His first symptom was a delay in motor development. He began walking independently at the age of 2.5 years, exhibiting a waddling gait with frequent falls and never attaining running or jumping abilities. By the age of 8 years, the patient became wheelchair-dependent. Due to limitations in independent mobility, he was transitioned to homeschooling from the second grade of school. Subsequently, progressive weakness in the arms developed, further hindering daily activities and academic performance. Episodic challenges with swallowing solid food began to emerge at the age of 9 years.

**Figure 1:**
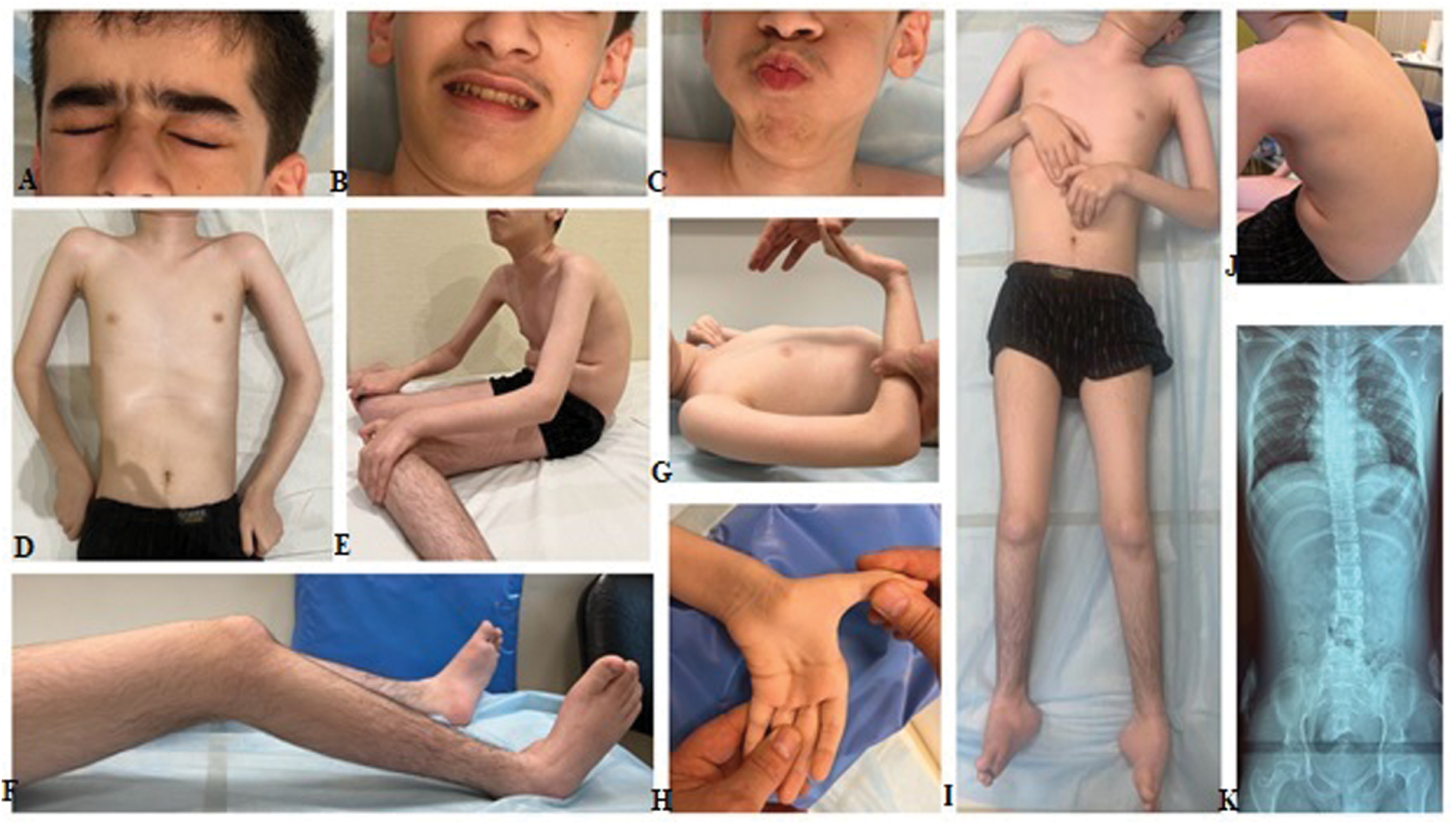
Clinical features of the proband include mild facial weakness observed during eye closure and cheek blowing **(A–C)**. Limb joints contractures, severe diffuse muscle atrophy, prominent kyphoscoliosis, and bilateral talipes varus are also noted **(D–J)**. Left-sided lumbar scoliosis and subluxation of the femoral heads are indicated **(K)**.

The clinical examination revealed a delay in physical development, with weight measuring 30 kg (−2.80 SD), stature 138 cm (−3.11 SD), and a head circumference of 57 cm (+1.55 SD). Mild weakness of the facial muscles and hypomimia were noted. However, swallowing functioned normally, with both pharyngeal and palatine reflexes intact. Phenotypic features included an arched palate, dolichocephaly, and protruding ears. Additionally, the patient exhibited pectus excavatum, cervical spine rigidity, and pronounced thoracolumbar kyphoscoliosis. Contractures were observed in the elbow, wrist, knee, and ankle joints, alongside ulnar deviation of the hands, hypermobility of the distal phalanges of the fingers, and pronounced bilateral pes cavus (Figure 1). Other findings included diffuse muscle hypotonia, muscle atrophy, diffuse areflexia, and notable weakness of the axial musculature. The patient was unable to lift his head from the couch or sit up independently from a prone position. Reduced cough reflex was attributed to weakness in abdominal and respiratory muscles. Muscle strength was significantly diminished in both proximal and distal leg muscles (0–1/5 on the MRC scale), as well as in proximal arm muscles (1–2/5) and distal arm muscles (3–4/5). Despite these limitations, the patient demonstrated some ability to hold cutlery and a mug. Severe restrictions in self-care necessitated constant external assistance, with wheelchair use being constant. At 14 years of age, the patient's creatine kinase (CK) level was determined for the first time and within the normal range at 96 U/L. Forced vital capacity measured at 82.8%. Cardiac ultrasound did not reveal any abnormalities, while x-rays of the spine and pelvis indicated first-degree left-sided lumbar scoliosis and subluxation of the femoral heads.

**Figure 2:**
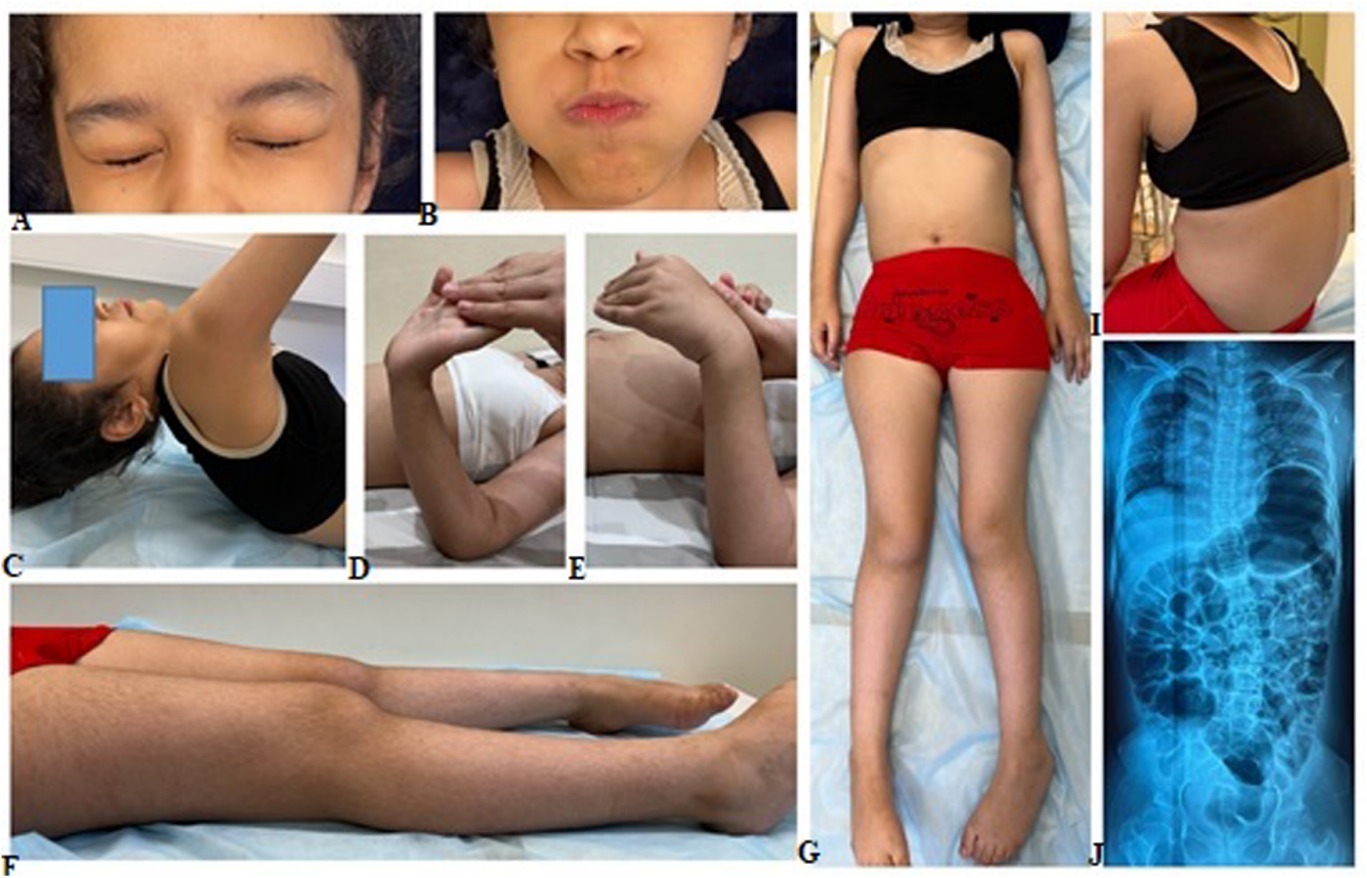
Clinical manifestation of the proband's sister included pronounced kyphoscoliosis, hypermobility of distal phalanges, varus deformity of the feet, shorter left leg, generalized hypotonia, and severe weakness in the muscles of the neck and lower limbs **(A–I)**. X-rays of the spine and pelvis shows left-sided lumbar scoliosis and subluxation of the femoral **(J)**.

The proband's sibling, a 10-year-old girl, was born during the third pregnancy with a birth weight of 3.3 kg (−0.24 SD). Like her brother, she experienced delayed motor development, achieving independent walking only after 2.5 years, and never displayed running or jumping behaviors. A gait disorder was observed at the age of 3 years. Due to the loss of independent walking ability and weakness in her arm muscles, the child has been homeschooled since the age of 8. At the age of 10, the girl underwent a clinical examination revealing normal physical development [weight 36 kg (+0.32 SD), stature 140 cm (+0.29 SD)] and relative macrocephaly [head circumference 57 cm (+3.61 SD)]. The girl relied on a wheelchair and displayed pronounced thoracolumbar kyphoscoliosis, hypermobility of the distal phalanges of the fingers, varus deformity of the feet, shortening of the left leg, diffuse muscle hypotonia, areflexia, and severe weakness in the neck and lower limb muscles (Figure 2). Like her brother, she also had a diminished cough reflex, indicating weakness in respiratory and abdominal muscles. While her upper limb muscles were less affected compared to her brother, her ability to perform self-care tasks was severely limited. Although she could briefly sit without support, she required constant assistance from caregivers. CK level was analyzed for the first time and were found to be normal at 114 U/L. Cardiac ultrasound examination revealed no abnormalities. X-rays of the spine and pelvis unveiled second-degree left-sided lumbar scoliosis and subluxation of the femoral heads.

Needle electromyography indicated myogenic changes in all tested muscles of both children without any pathological spontaneous activity in the proband and mild spontaneous activity in his sister.

**Figure 3:**
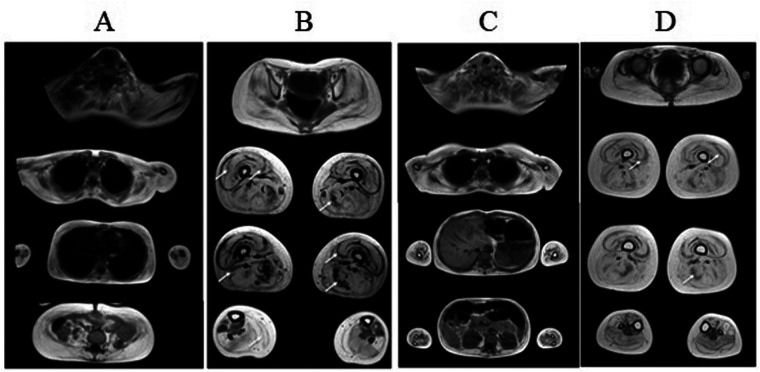
Whole body muscle MRI of proband **(A–B)** and proband's sister **(C–D)**. Axial T1-weighted imaging revealed similar patterns of fatty infiltration and muscle involvement in both children. The third and forth grade of fatty muscle infiltration in upper and lower limb muscles are noted. The cores of preserved muscle within the vastus lateralis (arrows), vastus medialis in both children (“sandwich”-like pattern), relative preservation of the semimembranosus, adductor magnus muscles (arrows) and tibialis posterior, extensor digitorum longus muscles.

In both children, whole body MRI revealed total fatty muscle replacement on the third and forth grade according to the four-point Mercuri scale in the axial T1-weighted imaging (Figure 3). The most spared muscles were neck muscles, anterior compartment of the forearm muscles, gluteus medius, tibialis posterior and extensor digitorum longus. Despite more severe clinical features in the proband, he had more preserved extensor digitorum longus muscles. Both patients exhibited the cores of preserved muscle within the vastus lateralis, vastus medialis, resembling “sandwich”-like pattern. It's worth noting the presence of proximo-distal gradient of relative preservation of the semimembranosus muscle with the same “sandwich”-like pattern and relative hypertrophy of semitendinous muscles with total fatty replacement of them. Muscle biopsy was not performed due to patients' refusal on religious reasons.

**Figure 4:**
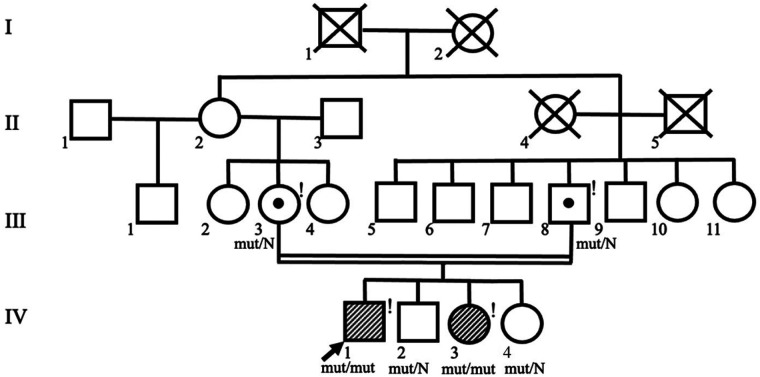
Pedigree of the family shows consanguinity of the proband's parents. Symbols of the proband (IV.1) and his affected sister (IV.3) are shaded. Exclamation marks indicate examined family members.

Whole-exome sequencing revealed a novel variant in the last exon 26 of the *JAG2* gene c.3467_3470dup, resulting in a frameshift and premature termination of translation (p.Pro1158AlafsTer22) in a homozygous state. The variant was classified as likely pathogenic according to ACMG criteria (PM2, PVS1) (10). The identified variant was confirmed in the proband and his sister by Sanger sequencing (the variant c.3467_3470dup inherited from the father and from the mother). According to the parents' examination, there were no symptoms of muscle weakness, cardiomyopathy, elevated CK level or delayed motor or speech development during childhood. When planning childbearing in the family prenatal diagnostics by Sanger sequencing of foetal DNA was carried out at the early stages of pregnancy. Heterozygous carriage of the c.3467_3470dup variant in the *JAG2* gene was found, indicating a favourable prognosis for the fetus, and a healthy girl was born into the family (IV.4) (Figure 4).

## 3 Materials and methods

Both patients underwent nerve conduction studies and needle electromyography of clinically affected muscles, including the tibialis anterior, vastus lateralis, deltoideus, and biceps brachii (DANTEC Keypoint, USA). Whole-body muscle magnetic resonance imaging (MRI), comprising axial T1-weighted and axial T2-STIR images, was performed using a 1.5 T magnetic resonance scanner (Philips Ingenia, the Netherlands).

The proband underwent whole-exome sequencing carried out using paired-end sequencing (2 × 75 bp) on an IlluminaNextSeq 500 sequencer (Illumina, San Diego, California, U.S.). The library was constructed using the KAPA Hyper Prep Kit (F. Hoffmann-La Roche Ltd, Switzerland). Target enrichment was performed with an IDT xGen® Exome Research Panel v.1 solution capture array (IDT inc., USA), including the coding regions of 19,396 known genes. The detected variant was annotated according to the HGVS (Human Genome Variation Society) nomenclature: https://hgvs-nomenclature.org/stable/recommendations/DNA/duplication/ (version 21.0.0) (HGVS, RRID:SCR_012989). The sequencing data were analyzed using the NGS-data-Genome program developed at the Department of Bioinformatics of FSBSI RCMG (registration number 2021662113). Mean coverage was ×80.9, with 0.55% of fragments with less than ×10 coverage.

The segregation analysis of the identified variant was conducted using Sanger sequencing performed on an ABIPrism 3,500 Genetic Analyzer (Applied Biosystems, Foster City, CA, USA), following the manufacturer's protocol. Primer sequences were designed, and the variant was designated according to the NM_002226.5 transcript variant.

## 4 Discussion

LGMD type R27 is a rare autosomal recessive disorder resulting from biallelic variants in the *JAG2* gene. This gene encodes the ligand of the Notch1 receptor, a crucial component of the highly conserved Notch signaling pathway (4). This pathway plays a pivotal role in intracellular signal transduction and the regulation of intercellular interactions during both embryonic development and postnatal tissue maintenance across various tissues. In current literature, there are only three articles delineate the clinical and genetic data of 27 patients diagnosed with LGMD type R27, stemming from 16 distinct families hailing from diverse geographical regions. All these studies unveiled twenty single nucleotide variants, with fifteen identified as missense substitutions in either the homozygous or compound heterozygous states (1–3). Notably, it is postulated that numerous of these documented missense variants disrupt the structural conformation of the Jagged2 protein (1).

In our study, we identified a novel homozygous variant, c.3467_3470dup (p.Pro1158AlafsTer22), in the *JAG2* gene in two siblings aged 14 and 10 years. This variant results in a frameshift mutation and premature termination of translation, leading to a truncated protein that is shorter compared to wild type by 60 amino acid residues with the replacement of 21 amino acid residues. Interestingly, previous research had suggested that all disease-causing variants in the *JAG2* gene exhibit a loss-of-function effect. However, it did not correspond well with the expected high intolerance for loss-of-function mutations in the gnomAD database v.2.1 (Pl = 1, and observed/expected ratio = 0.06) for an autosomal recessive gene. This underscores the need for further research to elucidate the molecular mechanisms underlying the pathogenicity of *JAG2* variants and their association with clinical phenotypes. Notably, performing whole exome sequencing in patients with genuine empty follicle syndrome a novel frameshift pathogenic variant (p. Ser1198ThrfsTer33) was found in the *JAG2* gene together with other variant in *HALPN1* gene (11). Authors suggest these two variants could be the cause of this disease because of Notch pathway also playing significant roles in follicle assembly and growth (12).

We report clinical features of two additional molecularly confirmed *JAG2*-related myopathy cases from a consanguineous family, and review the core phenotypic features of our cases and those observed in previously reported cases (Table 1). The disease exhibits a variable age of onset, ranging from infancy to adolescence, with patients presenting with symptoms of limb girle muscular dystrophy affecting the upper and lower extremities. Initial involvement typically targets the proximal leg muscles and neck flexors, followed by progressive involvement of the proximal upper limb muscles (1–3). Clinical picture in both children marked by widespread muscle hypotonia predominantly affecting the pelvic girdle and proximal leg muscles, accompanied by areflexia, contractures of major joints, and spinal scoliosis. All these features were previously described in most patients. Additionally, the patient had pectus excavatum and cervical spine rigidity, which was also observed in a third of patients in the Coppens et al. study (1). The disease exhibited prompt progression, resulting in the loss of independent ambulation by the age of 8 in both affected children. Approximately 10 of the 27 patients described loss their ambulation, who usually like our patients have early disease manifestation (1, 3). Conversely, when the disease manifests during adolescence or adulthood, progression tends to be slower.

**Table 1:** Clinical comparison of proband and proband's sister with previous *JAG2*-muscular dystrophy cases (1–3).

	Family 1 Present		Family 1 Dofash L 2024		Family 2 Dofash L 2024	Family 1 Schrama E 2023	13 families Coppens S 2021
	Patient 1 (Proband)	Patient 2 (Proband's sister)	Patient 1	Patient 2	Patient 1	Patient 1	23 patients
General							
Age (years)	14	10	8	6	5	36	From 5–53
Country (origin)	Tajikistan	Tajikistan	Pakistan	Pakistan	Europe	Dutch	Morocco, Iran, USA, Europe, UK, Sri Lanka, UAE, Egypt
Consanguinity	+	+	+	+	–	–	6 families/13
Gender	M	F	M	M	M	M	11F/12M
Molecular							
*JAG2* variants	c.3467_3470dup	c.3467_3470dup	c.1021G > T, p.(Gly341Cys)	c.1021G > T, p.(Gly341Cys)	c.703T > C p.(Trp235Arg)	c.1936C > T p.(Arg646Cys)	10 missense, 1 nonsense, 2 frameshift, 1 in-frame deletion, 14q32.33 deletion encompassing
	p.(Pro1158AlafsTer22)	p.(Pro1158AlafsTer22)					
					c.2350C > T p.(Arg784Cys)	c.2557T > G p.(Cys853Gly)	*JAG2*
Zygosity	Homozygous	Homozygous	Homozygous	Homozygous	Compound heterozygous	Compound heterozygous	Homozygous, Compound heterozygous
Clinical features							
Period of onset	Early childhood	Early childhood	Early childhood	Early childhood	Early childhood	Young adult	5 Infancy/
							9 Childhood/
							4 Young adult/
							5 Adolescence
Progression	Rapid	Rapid	Rapid	Rapid	Rapid	Slow	Rapid 7/23
Loss of ambulation (years)	8	8	AW at 8	6	6	No	8/23
PUL weakness	+	+	+	+	+	–	23/23
DUL weakness	+	+	–	–	+	–	15/23
PLL weakness	+	+	+	+	+	+	23/23
DLL weakness	+	+	–	–	+	–	18/23
Muscle atrophy	+	–	+	+	+	–	8/23
Contractures	+	+	+	+	+	–	13/23
	Elbows, wrists, knees, ankles	Ankles	Ankles	Knees, ankles	Hamstrings, ankles		
Facial weakness	+ mild	+ mild	+ mild	+ mild	ND	+ mild	4/23
Neck weakness	+ severe	+ severe	+	+	+	–	19/23
Spine deformity, scoliosis	+	+	–	+ mild	+ mild	–	12/23
	Thoracolumbar kyphoscoliosis	Thoracolumbar kyphoscoliosis					
	Pectus excavatum						
Rigid spine	+ neck	–	–	–	ND	–	7/23
Intellectual disability	–	–	–	+ mild	–	+	
Cardiac disease	–	–	ND	ND	ND	–	5/23
Low FVC	+	ND	–	ND	–	–	13/20
Laboratory and instrumental results							
Elevated CK level	–	–	–	–	+ mild	+ mild	10/22
EMG	+ myopathic	+ myopathic	ND	ND	ND	ND	Myopathic: 16/19
Muscle MRI	+ fatty infiltration, cores of preserved muscle	+ fatty infiltration, cores of preserved muscle	ND	+ fatty infiltration, cores of preserved muscle	+ fatty infiltration, cores of preserved muscle	+ pattern of intramuscular demarcations of fat and relatively spared muscle tissue	11/11
Muscle biopsy	ND	ND	ND	+	+	+	Abnormal: 14/14
				Dystrophic	Dystrophic	Myopathic changes, subsarcolemmal glycogen deposition	

F, female; M, male; USA, United States of America, UAE, United Arab Emirates; ND, non documented; AW, assisted walking; EMG, electromyography; FVC, forced vital capacity; PUL, proximal upper limb; DUL, distal upper limb; PLL, proximal lower limb; DLL, distal lower limb; MRI, magnetic resonance imaging.

Mild facial weakness revealed in our patients was described previously only in seven individuals with LGMD R27. Approximately 70% of patients eventually exhibited weakness and atrophy of the distal muscles of both the upper and lower extremities. Due to fast disease progression diffuse muscle atrophy, weakness and joint contructures were observed in both children indicating the severity of LGMD. Contractures of major joints, and less commonly, minor joints, develop in over half of affected individuals (1–3).

In cases with early disease onset, albeit less frequently observed during childhood and adolescence, respiratory function impairment may manifest as a reduction in forced vital capacity. Respiratory testing performed in the proband showed slightly reduction of the forced vital capacity parameter. Cardyomyopathy documented in some patients was not revealed in our patients. In both children, whole body muscle MRI revealed severe changes corresponding to the severity of the clinical picture and disclosed similar features to the cases described in previous studies (1–3).

## 5 Conclusion

This paper presents a description of the clinical and genetic features of two siblings from the Republic of Tajikistan who harbor a newly identified homozygous variant in the *JAG2* gene. This variant results in truncated protein with 21 amino acid substitution within the cytoplasmic domain of the Jagged2 protein. Considering the early onset and severe disabling nature of the disease, we propose that this identified amino acid substitution may disrupt the intracellular site of the Notch signaling pathway, thereby affecting disease pathogenesis. Early diagnosis and genetic counseling for prenatal planning are necessary due to the severity of LGMD R27 symptoms in some patients.

## Data Availability

The datasets presented in this study can be found in online repositories. The names of the repository/repositories and accession number(s) can be found in the article/Supplementary Material.
